# Thin Film Mixed Matrix Hollow Fiber Membrane Fabricated by Incorporation of Amine Functionalized Metal-Organic Framework for CO_2_/N_2_ Separation

**DOI:** 10.3390/ma14123366

**Published:** 2021-06-17

**Authors:** Guoqiang Li, Wojciech Kujawski, Katarzyna Knozowska, Joanna Kujawa

**Affiliations:** 1 Department of Physical Chemistry and Physical Chemistry of Polymers, Faculty of Chemistry, Nicolaus Copernicus University in Toruń, 7 Gagarina Street, 87-100 Toruń, Poland; 2 Moscow Engineering Physics Institute, National Research Nuclear University MEPhI, 31 Kashira Hwy, 115409 Moscow, Russia

**Keywords:** thin film hollow fiber membranes, amine functionalized nanoparticles UiO-66-NH_2_, mixed matrix membranes (MMMs), CO_2_/N_2_ separation

## Abstract

Membrane separation technology can used to capture carbon dioxide from flue gas. However, plenty of research has been focused on the flat sheet mixed matrix membrane rather than the mixed matrix thin film hollow fiber membranes. In this work, mixed matrix thin film hollow fiber membranes were fabricated by incorporating amine functionalized UiO-66 nanoparticles into the Pebax^®^ 2533 thin selective layer on the polypropylene (PP) hollow fiber supports via dip-coating process. The attenuated total reflection-Fourier transform infrared (ATR-FTIR), scanning electron microscope (SEM), energy-dispersive X-ray spectroscopy (EDX) mapping analysis, and thermal analysis (TGA-DTA) were used to characterize the synthesized UiO-66-NH_2_ nanoparticles. The morphology, surface chemistry, and the gas separation performance of the fabricated Pebax^®^ 2533-UiO-66-NH_2_/PP mixed matrix thin film hollow fiber membranes were characterized by using SEM, ATR-FTIR, and gas permeance measurements, respectively. It was found that the surface morphology of the prepared membranes was influenced by the incorporation of UiO-66 nanoparticles. The CO_2_ permeance increased along with an increase of UiO-66 nanoparticles content in the prepared membranes, while the CO_2_/N_2_ ideal gas selectively firstly increased then decreased due to the aggregation of UiO-66 nanoparticles. The Pebax^®^ 2533-UiO-66-NH_2_/PP mixed matrix thin film hollow fiber membranes containing 10 wt% UiO-66 nanoparticles exhibited the CO_2_ permeance of 26 GPU and CO_2_/N_2_ selectivity of 37.

## 1. Introduction

Global warming resulted from greenhouse gas has created serious consequence for the environment, e.g., melting glaciers. In comparison with other greenhouse gases, CO_2_ is one of the important contributors to global warming [1]. CO_2_ emission increases significantly every year due to the rapid development of industry and the more intensive human activities. The flue gas released by power plant due to the usage of fossil fuels is the main source of CO_2_ emission [2]. Therefore, the separation of CO_2_ from flue gas mixture to mitigate the CO_2_ emission plays an important role in the environment protection and the sustainable development of the industry [3]. Membrane separation technology, physical and chemical adsorption, and cryogenic separation have been used in the CO_2_ capture process [1,4,5].

Membrane separation technology is widely considered as an alternative to the traditional intensively energy-consuming technologies for CO_2_ separation [6]. Various types of membrane have been used for CO_2_ capture from flue gas mixture, such as polymeric membranes [7,8], inorganic membranes [9], and mixed matrix membranes (MMMs) [10,11,12].

Pebax^®^ materials are used for MMMs fabrication because of their advantages, e.g., desirable separation performance and high processability [13]. They are good candidates for polymeric membrane matrix for CO_2_/N_2_ separation due to their desirable CO_2_ permeance, high ideal selectivity, and tunability of gas separation properties via the incorporation of nanofillers [14]. Pebax^®^ 2533 shows higher CO_2_ permeability with desirable CO_2_/N_2_ selectivity [15]. Therefore, Pebax^®^ 2533 was chosen as the polymer matrix for the preparation of thin film mixed matrix hollow fiber membranes in this work. Thin film Pebax^®^ 2533/polyetherimide (PEI) composite hollow fiber membranes were fabricated via dip coating method and assembled into a lab-scale hollow fiber module for CO_2_/N_2_ separation [13]. In the pure gas permeance test, the prepared membranes exhibited CO_2_ and N_2_ permeances equal to 48 and 1.6 GPU, respectively, at 23 °C and 790 kPa, while the CO_2_ and N_2_ permeances are 36 and 1.7 GPU, respectively, in the gas mixture permeance test under the same testing conditions. The CO_2_ permeance from gas mixture test was 12 GPU lower than that from pure gas test. However, the N_2_ permeance in both cases are practically the same [13].

MMMs containing metal-organic framework (MOF) have been intensively studied to improve the comprehensive gas separation properties of membranes. This is because MOFs possess high surface area, high packing capacity, tunable porosity and pore size, chemical functionality, and enormous varieties, which endows them huge advantages for the incorporation into polymer matrix [16,17,18]. MOFs are more intensively used in MMMs for various gas separation processes than other porous fillers [5].

MOFs such as the zeolitic imidazolate framework (ZIF) [17,18,19], Materials Institute Lavoisier (MIL) [20], and University in Oslo (UiO-66) [21,22] are commonly used for the preparation of MMMs for gas separation. Gao et al. [19] incorporated ZIF-7-NH_2_, ZIF-7-OH, and ZIF-7-CH_3_OH into Pebax^®^ 2533 matrix to fabricate MMMs. The CO_2_ adsorption properties of MMMs was enhanced due to the introduction of functional groups in ZIF-7 framework. All the prepared MMMs showed better CO_2_/N_2_ separation performance than the pristine Pebax^®^ membranes. The MMM containing 14 wt% ZIF-7-OH particles exhibited high CO_2_ permeability equal to 273 Barrer and CO_2_/N_2_ selectivity equal to 38, which in comparison to the pristine Pebax^®^ membrane increased by 60 and 145%, respectively. Jameh et al. [23] modified ZIF-8 nanoparticles with ethylenediamine (ED) and incorporated them into Pebax^®^ 1074 matrix to fabricate MMMs for CO_2_ capture. The authors found that the CO_2_ adsorption capacity of MMM containing ED modified ZIF-8 was higher than that containing ZIF-8. Consequently, CH_4_ and CO_2_ permeabilities of the ZIF-8/Pebax^®^ MMMs are 9.39 and 134 Barrer, respectively, while for the ED-ZIF-8/Pebax^®^ MMMs, the CH_4_ and CO_2_ permeabilities were 14.2 and 344 Barrer, respectively. Dai et al. [24] incorporated ZIF-8 into polyetherimide (PEI) matrix to prepare dual layer mixed matrix hollow fiber membranes via dry jet-wet spinning technique. It was found that presence of ZIF-8 increased the CO_2_ permeance and CO_2_/N_2_ ideal selectivity from 13 GPU and 34 to 21 GPU and 39, respectively, in comparison to pure PEI hollow fiber membranes. Etxeberria-Benavides et al. [25] prepared polybenzimidazole (PBI) mixed matrix hollow fiber membranes containing ZIF-8 for H_2_/CO_2_ separation. The prepared membranes showed high H_2_ permeance of 107 GPU at 7 bar and 70 °C in comparison with 65 GPU of pristine PBI hollow fiber membranes. While the H_2_/CO_2_ selectivity was constant. For the mixed gas permeation, the improvement of H_2_/CO_2_ separation performance for PBI mixed matrix hollow fibers is hindered at high pressure around 30 bar because of the CO_2_ adsorption in ZIF-8, which blocks the H_2_ transport [25]. Song et al. [20] prepared Pebax^®^ 1657/MIL-101 and Pebax^®^ 1657/NH_2_-MIL-101 MMMs for CO_2_/N_2_ separation under sub-ambient condition. Authors found that the Pebax^®^ 1657/NH_2_-MIL-101 MMM possesses higher CO_2_/N_2_ selectivity equal to 95.6 comparing to CO_2_/N_2_ selectivity equal to 89.4 for Pebax^®^ 1657/MIL-101 MMM. This is because the amino-modified MIL-101 introduced –NH_2_ group possessing higher affinity to CO_2_. Therefore, the solubility and adsorption capacity of CO_2_ in MMMs were improved [20].

The preparation of flat sheet MMMs containing UiO-66 and UiO-66-NH_2_ for gas separation has been also studied [14,21,22]. Shen et al. [14] prepared UiO-66/Pebax^®^ 1657 and UiO-66-NH_2_/Pebax^®^ 1657 flat sheet MMMs for CO_2_/N_2_ separation. It was found that the UiO-66-NH_2_ nanoparticles showed higher affinity to carbon dioxide than UiO-66. The dispersibility of nanoparticles in the polymer matrix was improved due to the enhanced hydrogen bonding between fillers and polymer chains. With MOF loading of 10 wt%, UiO-66-NH_2_-Pebax^®^ 1657 MMM showed higher CO_2_/N_2_ selectivity and slightly lower CO_2_ permeability than those of UiO-66-Pebax^®^ 1657 membrane [14]. Chuah et al. [21] investigated CO_2_/N_2_ separation performance of polyimide-based MMMs containing UiO-66 possessing different functional groups (–HN_2_, –Br, –(OH)_2_). It was found that the functionalized UiO-66 in MMMs can effectively increase the CO_2_ diffusivity while suppressing N_2_ adsorption [21]. In the above examples, the UiO-66 nanoparticles with various functional groups are synthesized by using pre-synthetic functionalization. It is believed that the further functionalization of UiO-66-NH_2_ by using post-synthetic functionalization method can further tune the properties of UiO-66-NH_2_, such as the CO_2_ affinity and adsorption capacity, pore size, and surface area. Consequently, the CO_2_ capture ability of MMMs is enhanced [22]. Jiang et al. [22] modified UiO-66-NH_2_ with imidazole-2-carbaldehyde (ICA) via amine condensation. After modification, the pore volume and BET (Brunauer-Emmett-Teller) area of UiO-66-NH_2_ were reduced while the CO_2_ affinity and CO_2_/CH_4_ adsorption selectivity were increased. It was found that when 10 wt% modified UiO-66-NH_2_ was incorporated into Matrimid^®^ membranes, the high CO_2_/CH_4_ selectivity of 64.7 was obtained, which is 40% higher than the membranes containing UiO-66-NH_2_. As aforementioned, the presence of amine groups improved the CO_2_ adsorption capacity for UiO-66-NH_2_, resulting in higher CO_2_ solubility of MMMs, consequently, the higher CO_2_/N_2_ selectivity. Moreover, UiO-66-NH_2_ shows high compatibility with polymer matrix due to the hydrogen bonding between Pebax^®^ chains and UiO-66-NH_2_. Hence, UiO-66-NH_2_ was incorporated into Pebax^®^ 2533 matrix to improve the gas separation properties of thin film mixed matrix hollow fiber membranes in this work.

Microporous polypropylene hollow fiber membranes are suitable to be used as a support for the fabrication of composite hollow fiber membranes due to their desirable properties, e.g., high void volumes, well-controlled porosity, chemical inertness, good mechanical strength, and low cost [26]. Therefore, polypropylene hollow fibers were used as supports for the preparation of thin film mixed matrix hollow fiber membranes in this work.

Hollow fiber membranes have a promising future in various gas separation processes due to their advantages, e.g., high packing density and a self-supporting structure [6,7]. However, the flat sheet MMMs have been intensively studied since the incorporation of fillers, e.g., MOF particles can significantly enhance the gas separation performance of polymeric membranes. Therefore, it is highly necessary to investigate the formation of a selective layer containing filler e.g., MOF particles on the hollow fiber support and their gas separation performance. In this work, the main objective is to develop thin film mixed matrix hollow fiber membranes by incorporating UiO-66-NH_2_ filler into the Pebax^®^ 2533 selective layer. The effect of UiO-66-NH_2_ filler on the morphology, surface chemistry, and CO_2_/N_2_ separation performance of the prepared thin film mixed matrix hollow fiber membranes will be investigated.

## 2. Experimental

### 2.1. Materials

Polypropylene (PP) hollow fibers were kindly provided by Faculty of Chemical and Processing Engineering, Warsaw University of Technology (Warsaw, Poland). The PP hollow fibers were prepared via a dry-jet-wet spinning process, using a laboratory made setup. The PP hollow fibers possessed outer diameter of 2.6 mm, internal diameter of 1.8 mm, average pore size of 0.3 µm, and porosity of 50–60% [27]. Pebax^®^ 2533 was provided by Arkema (Colombes, France). Pebax^®^ 2533 consists of poly(ethylene oxide)—PEO block (80 wt%) and polyamide—PA-12 block (20 wt%).

Zirconylchloride octahydrate was supplied by Acbr GmbH (Karlsruhe, Germany). 2-aminoterephthalic acid and N,N-dimethylformamide (DMF) were purchased from Sigma Aldrich (Poznań, Poland). Ethanol was purchased from Alchem Grupa Sp. z o.o. (Toruń, Poland). CO_2_ (99.999%) and N_2_ (99.999%) were purchased from Air Products Sp. z o.o. (Siewierz, Poland). The mixing nozzles and epoxy resin were delivered by Farnell (Warsaw, Poland).

### 2.2. UiO-66-NH_2_ Synthesis

To synthesize UiO-66-NH_2_, 4.34 g of 2-aminoterephthalic acid and 7.6 g of zirconylchloride octahydrate were added into 72 mL of DMF. The homogeneous mixture was obtained by stirring (Heating magnetic stirrer, VELP Scientifica, Usmate Velate, Italy) and sonication (BANDELIN SONOREX, BANDELIN electronic GmbH & Co. KG, Berlin, Germany) at room temperature. Then the homogeneous mixture was kept in oven (Memmert GmbH + Co. KG, Schwabach, Germany) at 120 °C for 24 h. Then, the temperature of the mixture decreased to room temperature and centrifuged (High speed centrifuge type 310, Mechanika Precyzyjna, Warsaw, Poland) at 5000 rpm for 15 min. The obtained products were washed three times with DMF and ethanol, respectively. Finally, the obtained products were dried at room temperature and further in the oven at 150 °C for 4 h.

### 2.3. Fabrication of Pristine Pebax/PP and Pebax^®^ 2533-UiO-66-NH_2_/PP Thin Film Mixed Matrix Hollow Fiber Membranes

**Figure 1:**
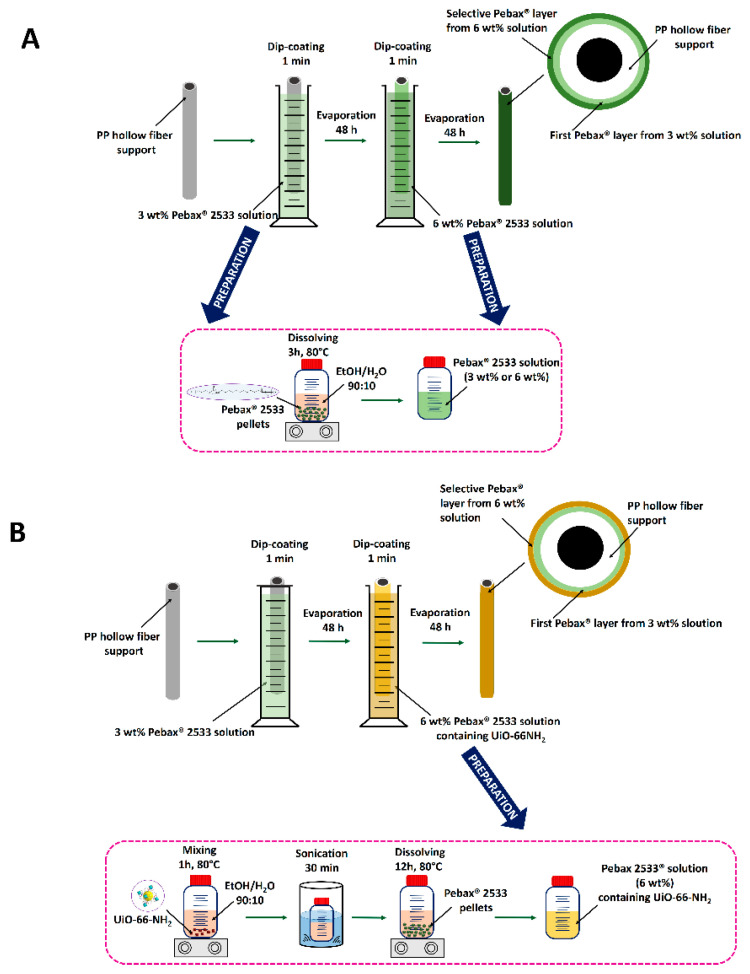
Schematic illustration of the preparation pristine Pebax^®^ 2533 thin film hollow fiber membrane (**A**) and Pebax^®^ 2533-UiO-66-NH_2_/PP mixed matrix thin film hollow fiber membrane (**B**).

To prepare the pristine Pebax^®^ 2533/PP thin film hollow fiber membranes, Pebax^®^ 2533 pellets were added into ethanol (90 wt%)/water (10 wt%) solvent. After that, the mixture was stirred at 80 °C for 3 h to obtain 3 and 6 wt% polymer solutions. Then, Pebax^®^ 2533 solution was cooled down to 25 °C. The dip-coating technique was used for the preparation of the thin Pebax^®^ 2533 layer on the shell side of the PP hollow fiber supports. First of all, a 10 cm long PP hollow fiber was prepared, and one end of the hollow fiber was sealed with epoxy resin. After the solidification of epoxy resin, the other end of the hollow fiber was attached to a metal holder. Then the single PP hollow fiber was vertically immersed into the 3 wt% Pebax^®^ 2533 solution for 1 min at room temperature. Finally, the Pebax^®^ 2533 coated hollow fiber was slowly taken from the coating solution and dried in air for more than 48 h for solvent evaporation. Afterwards, the second Pebax^®^ 2533 thin layer was formed from 6 wt% Pebax^®^ 2533 solution by using the same dip coating procedure. The preparation of the pristine Pebax^®^ 2533 thin film hollow fiber membrane was schematically illustrated in Figure 1A. The ideal selectivity of Pebax^®^ 2533/PP hollow fiber membranes fabricated by a single layer coating of 3 or 6 wt% Pebax^®^ 2533 solution was very low (Table S1), indicating the formation of defective Pebax^®^ 2533 layer. Therefore, a two-step coating process by using two different concentrations of Pebax^®^ 2533 solution was applied in this research.

To prepare the Pebax^®^ 2533-UiO-66-NH_2_/PP thin film mixed matrix hollow fiber membranes, a proper amount of UiO-66-NH_2_ particles were firstly dispersed into the ethanol/water solution (90:10 wt%/wt%) under continuous stirring for 1 h at 80 °C. Then 30 min sonication process was applied to the UiO-66-NH_2_ suspension for better dispersion of UiO-66-NH_2_ particles. Afterwards, 10% of the required amount of Pebax^®^ 2533 pellets was added to solvent mixture under continuous stirring for 2 h at 80 °C. Finally, the rest of Pebax^®^ 2533 pellets was dissolved into the solvent mixture under continuous stirring for 12 h at 80 °C to obtain 6 wt% Pebax^®^ 2533 solution containing UiO-66-NH_2_. The dip coating process for the preparation of Pebax^®^ 2533-UiO-66-NH_2_/PP thin film mixed matrix hollow fiber membranes is the same as for the preparation of pristine Pebax^®^ 2533/PP thin film hollow fiber membranes. The first layer was formed on the PP hollow fiber support from 3 wt% Pebax^®^ 2533 solution, and the second layer was formed from the 6 wt% Pebax^®^ 2533 solution containing UiO-66-NH_2_. The fabrication of the Pebax^®^ 2533-UiO-66-NH_2_/PP mixed matrix thin film hollow fiber membrane was schematically illustrated in Figure 1B.

### 2.4. Characterization

The morphology and element mapping of the UiO-66-NH_2_ particles, PP hollow fibers, Pebax^®^ 2533/PP and mixed matrix Pebax^®^ 2533/PP thin film hollow fiber membranes were analyzed by using scanning electron microscope (SEM) with X-ray spectroscopy (EDX) analysis—Phenom, Generation 5 (Phenom-Word B. V., Eindhoven, The Netherlands). The hollow fiber membranes were fractured in liquid nitrogen (Air Products, Siewierz, Poland) to prepare the samples for the cross-section SEM analysis. The Pebax^®^ 2533 layer thickness was measured on SEM pictures by using ImageJ software (version 1.8.0_172, 2020, University of Wisconsin, Madison, WI, USA).

The surface chemistry of UiO-66-NH_2_ particles, Pebax^®^ 2533/PP and mixed matrix Pebax/PP thin film hollow fiber membranes were analyzed by using FTIR-ATR spectroscopy. The FTIR-ATR spectra were obtained between 500 and 4000 cm^−1^ by using spectrometer Nicolet iS10 (Thermal Scientific, Waltham, MA, USA). The transmission mode with resolution of 4 cm^−1^ and 256 scans was applied. The obtained data was analyzed by Omnic 9 software (Version 9.2, 2012, Thermo Fisher Scientific, Waltham, MA, USA).

The TGA-DTA analyses for UiO-66-NH_2_ particles were conducted by using TA Instrument type SDT 2960 (TA Instrument, Champaign, IL, USA). The measuring temperature was set in the range of 25–950 °C under nitrogen atmosphere. The heating rate was 10 °C/min. The obtained data were analyzed by using TA Universal Analysis software (version: v5.5.24, 2015, TA Instrument, Champaign, IL, USA).

XRD analyses for UiO-66-NH_2_ particles were conducted by using Philips X”Pert (Malvern Panalytical, Malvern, UK). The transmission mode was applied. The measured 2θ range was in the range of 5−80°. The X’Celerator Scientific detector (Malvern Panalytical, Malvern, UK) with Cu anode was used.

The nitrogen adsorption/desorption measurements were conducted at −195.7 °C via Gemini VI (Micromeritics Instrument Corp., Norcross, GA, USA). All samples were degassed for 6 h at 110 °C before the measurements. The BET (Brunauer–Emmett–Teller) model was applied for the calculation of surface area.

### 2.5. Gas Permeance Measurements

To measure the gas permeance of hollow fiber membranes, the hollow fiber membranes should be assembled into the module. The module used for the gas permeance measurements of hollow fiber membranes was designed and assembled by the Membranes and Membrane Techniques Research Group in Nicolaus Copernicus University in Toruń, Toruń, Poland. All parts of the module were purchased from Swagelok (Toruń, Poland) (Figure S1). One hollow fiber with a length of 7–10 cm was assembled into the module. A potting process is needed before the assembling of hollow fiber membrane into the module. The details related to the set-up for gas permeance measurements, and the potting process are described elsewhere [7].

The pure gas (N_2_ and CO_2_) permeance tests were conducted at 2 bar and 25 °C. Each sample was measured 3 times under stabilized condition for better accuracy. The gas flow rate was measured by using a bubble flow meter (Sigma Aldrich, Poznań, Poland). The permeances (P/d) of gases were calculated by using Equation (1) [7,8]:

(1) $$\frac{P}{d}=\frac{Q}{\Delta \mathrm{pA}}=\frac{Q}{2n\pi \mathrm{rl}\Delta p}$$

where P is the permeability (Barrer); Q is the flux of gas permeation rate (cm^3^ (STP)/s); d is the thickness of membrane selective layer (cm); A is the effective membrane area (cm^2^); Δp is the pressure difference across the membrane (cmHg); r is the outer radius (cm) of hollow fiber; n is the number of hollow fibers; P/d is the gas permeance expressed in GPU (1 GPU = 10^−6^ cm^3^ (STP) cm^−2^ s^−1^ cmHg^−1^).

The ideal selectivity α was calculated by using Equation (2) [7,8]:

(2) $$\alpha_{12}=\frac{{\left(P/d\right)}_{1}}{{\left(P/d\right)}_{2}}=\frac{P_{1}}{P_{2}}$$

## 3. Results and Discussion

### 3.1. Characterization of UiO-66-NH_2_

**Figure 2:**
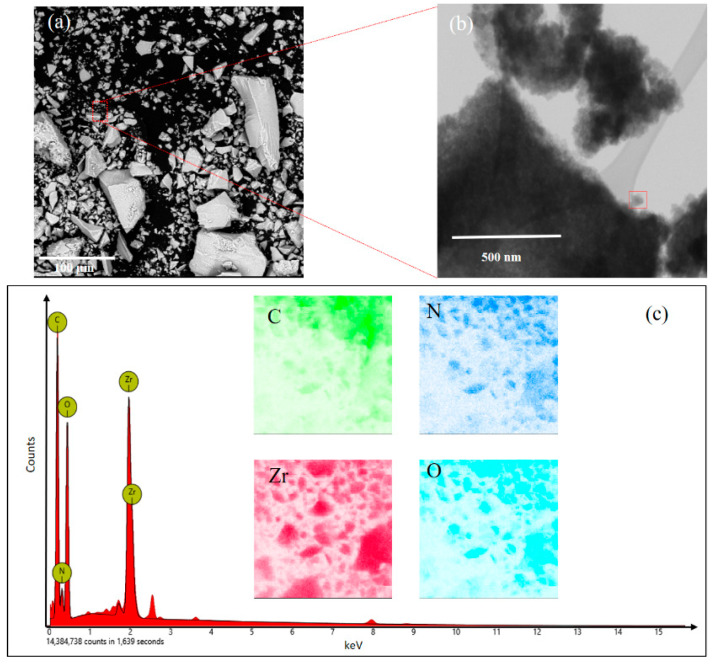
SEM (scanning electron microscope) images of synthesized UiO-66-NH_2_ particles (**a**,**b**). (**c**) is the corresponding EDX (energy-dispersive X-ray spectroscopy) data and mapping results of the selected area in (**a**) of UiO-66-NH_2_ particles.

The SEM and EDX results of the synthesized UiO-66-NH_2_ crystals were shown in Figure 2. The UiO-66-NH_2_ showed octahedrally rectangular shapes [14]. The particle size of UiO-66-NH_2_ is around 50–80 nm (Figure 2b). The elemental composition of synthesized UiO-66-NH_2_ was revealed by EDX analysis. UiO-66-NH_2_ is composed of Zr, C, O, and N elements (Figure 2c). The EDX results are in good agreement with its crystal structure which consists of Zr_6_-cluster and 2-aminoterephthalic acid linker.

**Figure 3:**
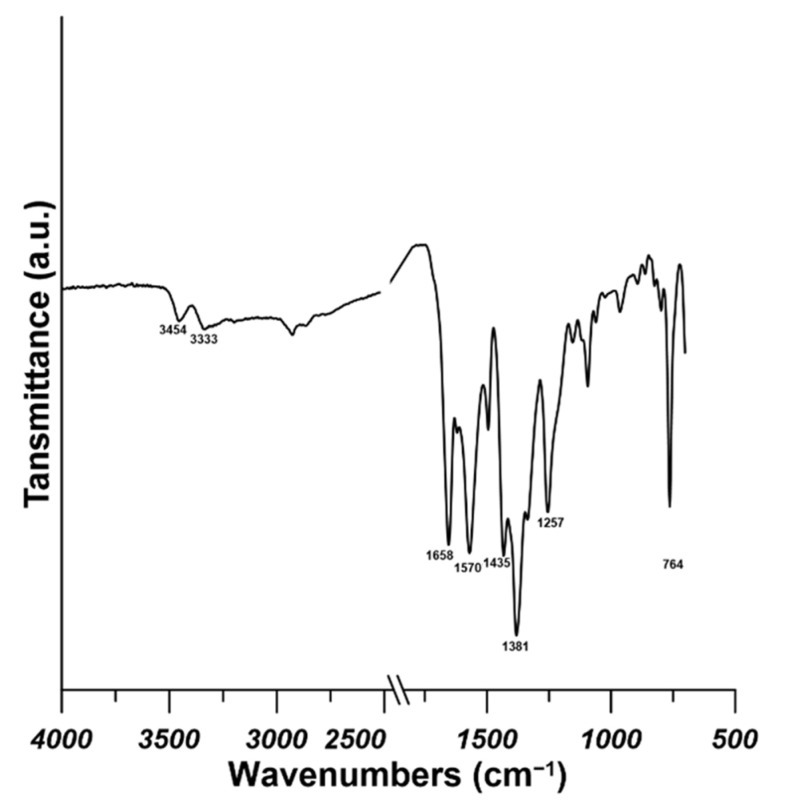
FTIR (fourier transform infrared) spectra of UiO-66-NH_2_.

As it is shown in Figure 3, the FTIR spectra provided more information about the chemical structure of the prepared UiO-66-NH_2_ particles. The intensive peak at 1658 cm^−1^ is ascribed to the stretching vibration of C=O group from residual DMF solvent in the MOF structure [28]. Two characteristic peaks at 3454 and 333 cm^−1^ can be ascribed to the asymmetric and symmetric stretching vibration of the primary amine group, respectively [29]. Moreover, the peak at 1620 cm^−1^ can be ascribed to the N–H bending vibration. What is more, the C–N bonding can be observed at 1257 and 1336 cm^−1^ due to the stretching vibration of C–N bond. The peak at 764 cm^−1^ can be assigned to the stretching vibration of Zr–O bond. The peak at 1435 cm^−1^ can be related to the C–C stretching vibration in the aromatic ring from the 2-aminoterephthalic acid ligand. Moreover, the peaks at 1381 and 1570 cm^−1^ can be assigned to the symmetric and asymmetric C–O stretching bonds, respectively, resulting from aromatic and carboxylic groups [30].

**Figure 4:**
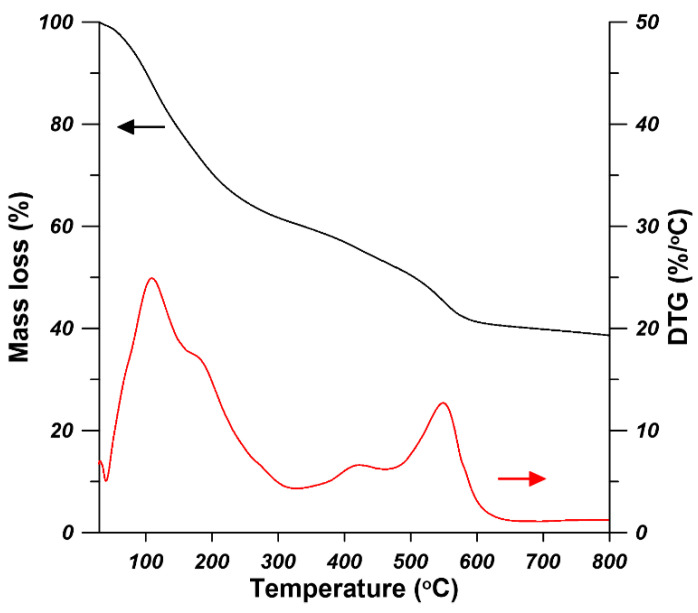
TGA (thermal gravimetric analysis) curves of UiO-66-NH_2_.

**Figure 5:**
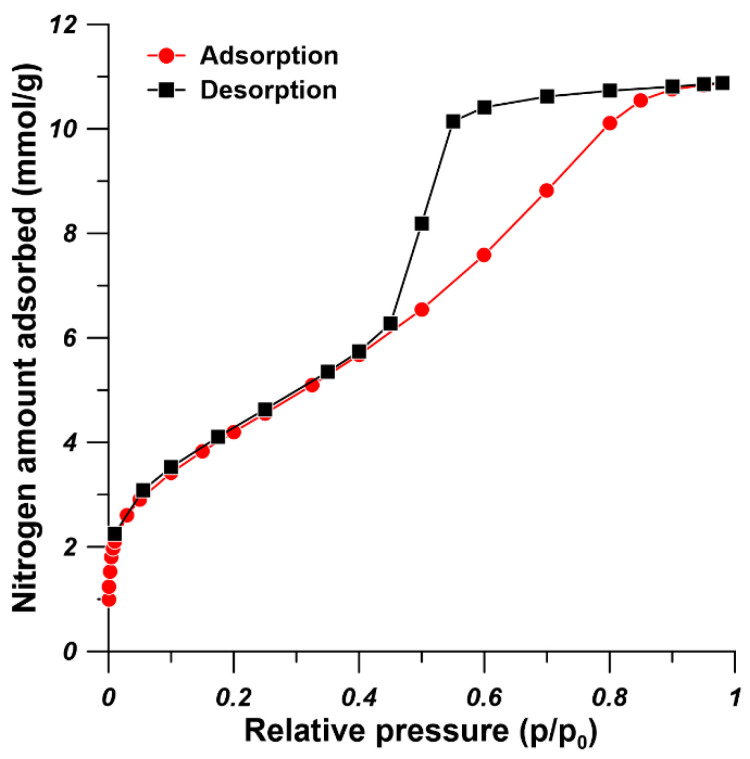
N_2_ adsorption and desorption curves of UiO-66-NH_2_.

The TGA and the DTG curves are presented in Figure 4. The DTG curve was plotted as a function of temperature since it can clearly provide information of the transitions of UiO-66-NH_2_. As it is shown in Figure 4, the TGA and DTG curves of UiO-66-NH_2_ show a two-step mass loss. The UiO-66-NH_2_ powder underwent fist-stage mass loss when the temperature increased to 280 °C. This is because the removal of absorbed moisture, residual solvent and the dehyroxylation of the Zr_6_O_4_(OH)_4_ into Zr_6_O_6_ [28]. The mass lass at this stage is around 40%. The crystal framework decomposition temperature for UiO-66-NH_2_ is around 380 °C indicated by the second-stage mass loss. At the second stage of mass loss, the decomposition of amino terephthalic acid ligand occurred and ZrO_2_ was formed [14]. Finally, when the temperature arrived at 650 °C, UiO-66-NH_2_ nanoparticles showed the largest mass loss around 68%. Cao et al. [31] also found that the decomposition of amino terephthalic acid ligand in UiO-66-NH_2_ nanoparticles occurred from 380 °C. When the temperature reaches 650 °C, the UiO-66-NH_2_ has the largest mass loss of approximately 65% [31]. The N_2_ adsorption-desorption isotherm measured at 77 K was used to determine the specific area and pore structure of UiO-66-NH_2_ (Figure 5). The adsorption hysteresis was observed due to the network effects and various forms of pore blocking [32], which could have resulted from the high increasing rate of temperature during the synthesis process. The BET (Brunauer-Emmett-Teller) surface area, adsorption average pore diameter, and BJH (Barrett–Joyner–Halenda) pore volume of the synthesized UiO-66-NH_2_ were 349.35 m^2^/g, 2.35 nm, and 0.49 cm^3^/g, respectively. Our results are in good agreement with the earlier reports [33,34].

**Figure 6:**
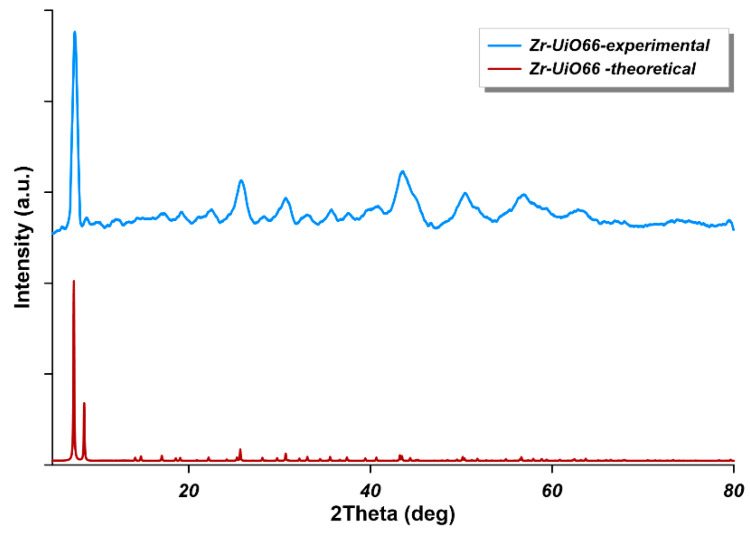
XRD (X-Ray diffraction) pattern of UiO-66-NH_2_ (the blue curve) and the theoretical XRD pattern of UiO-66 MOF (metal-organic frameworks) (the red curve).

The XRD analysis has been performed to prove that UiO-66-NH_2_ has been successfully synthesized. The formation of MOF was evidenced by the observation of characteristic intensive peaks at 2Theta equal to 7.5° (111) and 8.8° (002) (Figure 6). The experimental results are in a good accordance to the theoretical diffractogram, calculated based on the single crystal data (Figure 6) (ref code: SURKAT, deposit nr: 1405751) [35] with the implementation of Mercury software (Mercury 4. 2. 0., 2019, Cambridge Crystallographic Data Centre, Cambridge, UK).

### 3.2. Membrane Characterization

**Figure 7:**
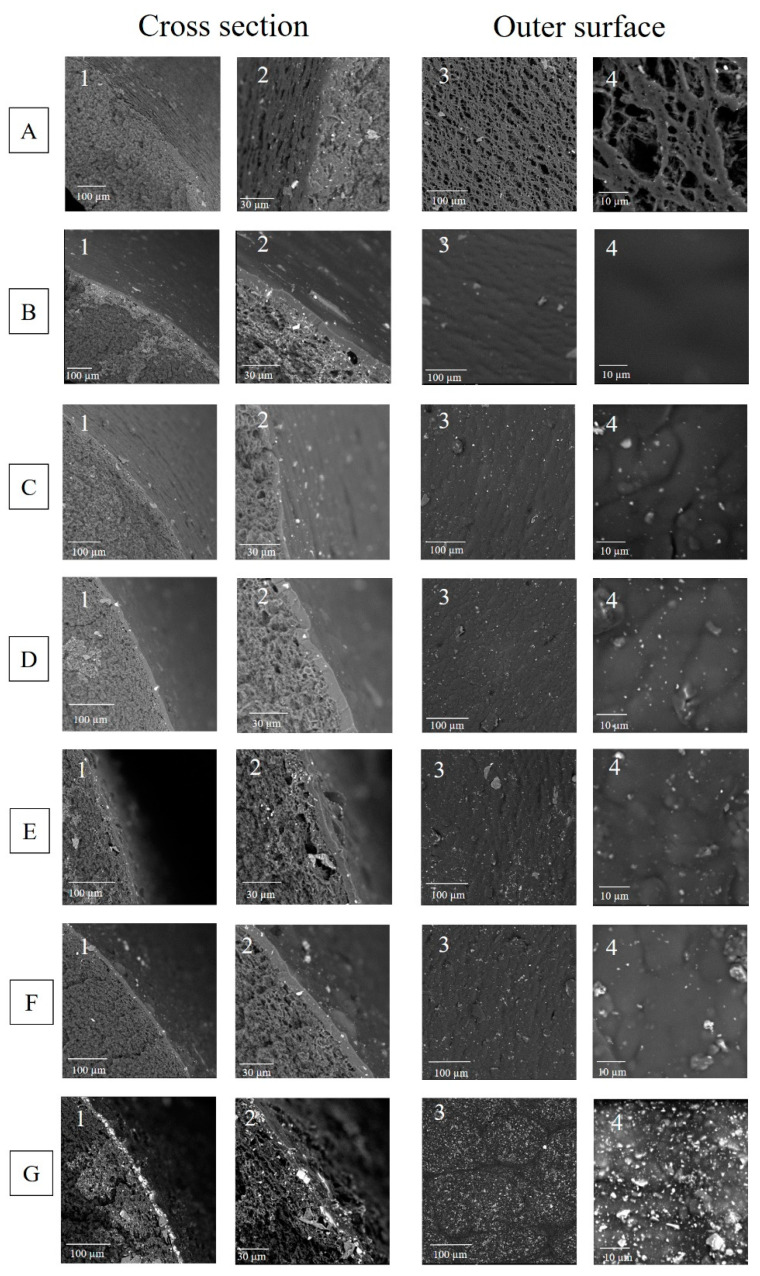
SEM pictures of cross-section (1 and 2) and outer surface (3 and 4) of Pebax^®^ 2533-UiO-66-NH_2_/PP thin film mixed matrix hollow fiber membranes—(**A**) PP hollow fiber support, (**B**) 0 wt% UiO-66-NH_2_, (**C**) 5 wt% UiO-66-NH_2_, (**D**) 10 wt% UiO-66-NH_2_, (**E**) 15 wt% UiO-66-NH_2_, (**F**) 20 wt% UiO-66-NH_2_, and (**G**) 50 wt% UiO-66-NH_2_.

The cross-section SEM and surface images of the polypropylene (PP) hollow fiber support, pristine Pebax^®^ 2533 membrane, and thin film mixed matrix hollow fiber membranes were shown in Figure 7. The Pebax^®^ 2533 thin layer was successfully coated on the shell side of PP hollow fibers by using a dip-coating method. As it is shown in Figure 7A, the PP hollow fiber supports possess porous structure (A1 and A2) and porous outer surface (A3 and A4). The porosity of PP hollow fiber is 50–60% and the average pore size is 0.3 µm [27]. After dip-coating with 3 and 6 wt% Pebax^®^ 2533 solutions, a defect-free Pebax^®^ 2533 selective layer was fabricated on the shell side of PP hollow fiber support (Figure 7B). When UiO-66-NH_2_ was incorporated into the Pebax^®^ 2533 matrix, the thin film mixed matrix membrane was successfully formed on the outer surface of PP hollow fiber support (Figure 7C–G). The loading amount of UiO-66-NH_2_ nanoparticles did not influence the thickness of Pebax^®^ 2533-UiO-66-NH_2_ hybrid selective layer since the coating Pebax^®^ 2533 solution was kept constant at 6 wt%. The thickness of the Pebax^®^ 2533-UiO-66-NH_2_ hybrid selective layer was in the range of 5.40–6.97 µm (Table S2). When comparing the morphology of the prepared hollow fiber membranes, the roughness of the shell side increased with the increase of the UiO-66-NH_2_ content from 0 to 50 wt% (Figure 7B4–G4). When the content of UiO-66-NH_2_ was low (5 and 10 wt%), the homogeneous dispersion of UiO-66-NH_2_ particles into Pebax^®^ 2533 matrix was observed (Figure 7B,C). At the high content of MOF particles (15, 20, and 50 wt%), the aggregation of UiO-66-NH_2_ in the polymeric matrix was observed (Figure 7E–G and Figure S2). It is reported that the MOF aggregation in polymeric matrix could lead to the formation of non-selective defects during the fabrication process [36]. Similar phenomenon was observed by Sutrisna et al. [33]. In their work, Pebax^®^ 1657-UiO-66/PVDF thin film mixed matrix hollow fiber membranes were prepared for CO_2_ separation. When the filler content was in the range of 10–50 wt%, no significantly aggregation was observed. However, the significant UiO-66 particle aggregation was observed when 80 wt% of UiO-66 was incorporated into the Pebax^®^ 1657 matrix [33].

**Figure 8:**
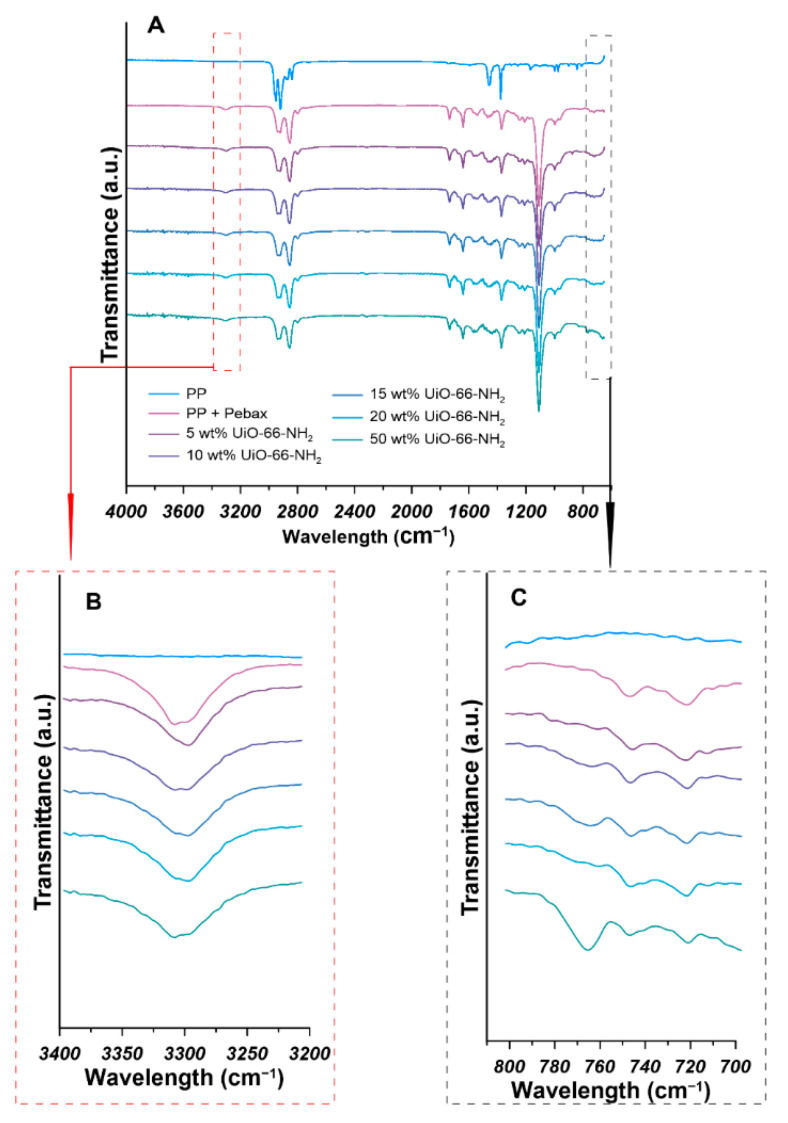
FTIR (Fourier transform infrared) spectra of Pebax^®^ 2533-UiO-66-NH_2_/PP thin film mixed matrix hollow fiber membranes (**A**) (the enlarged FTIR spectra in the wavelength range 3400–3200 cm^−1^ and 800–700 cm^−1^ are shown in (**B**) and (**C**), respectively).

To investigate the chemical structure of pure PP hollow fiber support and the prepared thin film mixed matrix hollow fiber membranes, FTIR analysis was conducted. The FTIR spectra of PP hollow fiber support and the prepared Pebax^®^ 2533-UiO-66-NH_2_/PP thin film mixed matrix hollow fiber membranes in the range of 650–4000 cm^−1^ were shown in Figure 8. As the FTIR spectra of PP shows, the peak at 841 cm^−1^ was attributed to C–CH_3_ stretching vibration. The peaks at 973, 998, and 1168 cm^−1^ were attributed to –CH_3_ rocking vibration. The symmetric bending vibration of –CH_3_ group was observed at 1376 cm^−1^. The –CH_3_ asymmetric stretching vibration was observed at 2951 cm^−1^. Besides the peaks related to methyl group in PP, the peaks at 1456, 2839, and 2919 cm^−1^ are designated to –CH_2_– symmetric bending, –CH_2_– symmetric stretching and –CH_2_– asymmetric stretching, respectively. Our FTIR results are in good agreement with the literature values [37,38]. After the formation of Pebax^®^ 2533 layer on the shell side of PP hollow fiber support, the characteristic peaks of the –CH_3_ group from PP disappeared. The characteristic peaks at 1109, 1640, 1734, and 3308 cm^−1^, are assigned to the stretching vibration of the C–O–C group of the PEO segment part, the N–H–C=O stretching vibration, the –O–C=O group, and the –N–H– stretching vibration of the polyamide block in Pebax^®^ 2533, respectively [33,39]. The FTIR spectra of the prepared Pebax^®^ 2533-UiO-66-NH_2_/PP thin film mixed matrix hollow fiber membranes are similar to the FTIR spectra of pristine Pebax^®^ 2533/PP hollow fiber membranes, which demonstrates that there were no strong chemical interaction between UiO-66-NH_2_ fillers and Pebax^®^ 2533 matrix. It was found that the red shift of FTIR characteristic peak related to the –N–H– stretching vibration occurred when the UiO-66-NH_2_ particles were incorporated into Pebax^®^ 2533 due to the formation of hydrogen bonding [14,40]. However, the peaks related to the –N–H– stretching vibration for the mixed matrix membrane containing 0, 5, 10, 15, 20, and 50 wt% of UiO-66-NH_2_ are 3308, 3297, 3297, 3296, 3307, and 3307 cm^−1^, respectively (Figure 8A). The –N–H– peak shift for PA (polyamide) segment is negligible due to the difficulty in the thin composite layer characterization [33]. As it is shown in the FTIR spectra in the range of 700–800 cm^−1^ (Figure 8C), a peak related to the stretching vibration of Zr–O bond around 764 cm^−1^ was observed, which indicates the serious aggregation of UiO-66-NH_2_ particles in the mixed matrix hollow fiber membranes. This finding is in good agreement with the SEM results (Figure 7G).

### 3.3. The Effect of UiO-66-NH_2_ Loading on Gas Separation Performance

**Figure 9:**
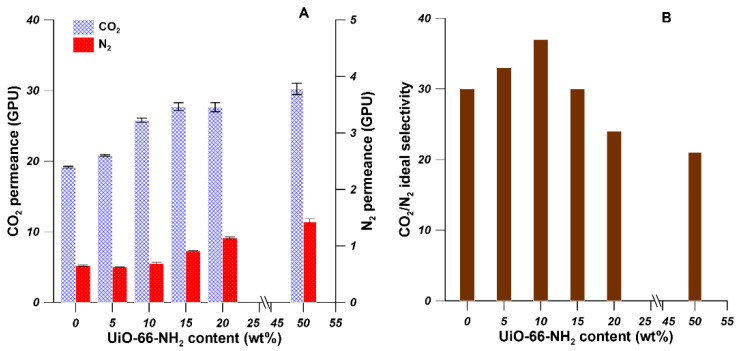
The effect of UiO-66-NH_2_ content on the CO_2_ and N_2_ permeances (**A**) and CO_2_/N_2_ ideal selectivity (**B**) of Pebax^®^ 2533-UiO-66-NH_2_/PP thin film mixed matrix hollow fiber membranes.

The gas separation behaviors of the prepared thin film mixed matrix hollow fiber membranes were studied by the gas permeation measurements. The CO_2_ and N_2_ permeance through the prepared membranes were measured at 2 bar and 25 °C. As it is shown in Figure 9, the UiO-66-NH_2_ content in the Pebax^®^ 2533 matrix influenced the gas permeance and the ideal selectivity of Pebax^®^ 2533-UiO-66-NH_2_/PP thin film mixed matrix hollow fiber membranes. As can be seen from Figure 9A, when the UiO-66-NH_2_ content increased from 0 to 50 wt%, the CO_2_ permeance increased significantly from 19 to 30 GPU. The N_2_ permeance barely increased when the UiO-66-NH_2_ content increased from 0 to 10 wt%. However, the N_2_ permeance increased to 0.91, 1.14, and 1.42 GPU when the UiO-66-NH_2_ content increased to 15, 20, and 50 wt%, respectively. As it is shown in Figure 9B, the CO_2_/N_2_ ideal selectivity firstly increased from 30 to 37 when the UiO-66-NH_2_ content increased from 0 to 10 wt%. Then the CO_2_/N_2_ ideal selectivity decreased to 21 when the UiO-66-NH_2_ content increased to 50 wt%.

As discussed above, when the UiO-66-NH_2_ content increased to 10 wt%, both the CO_2_ permeance and CO_2_/N_2_ ideal selectivity increased while the N_2_ permeance was practically unchanged, which indicates the formation of defect-free thin mixed matrix membrane on the PP hollow fiber support. The enhanced CO_2_ permeance and CO_2_/N_2_ ideal selectivity were ascribed to the interrupted chain packing in the polymer matrix [41] and the CO_2_-philic nature of UiO-66-NH_2_ [42]. In comparison to the pure Pebax^®^ 2533 thin film hollow fiber membrane, the CO_2_ permeance and CO_2_/N_2_ ideal selectivity of Pebax^®^ 2533-UiO-66-NH_2_/PP thin film mixed matrix hollow fiber membrane containing 10 wt% UiO-66-NH_2_ increased by 35 and 23%, respectively. The kinetic diameters for CO_2_ and N_2_ molecules are 0.33 and 0.36 nm, respectively. The CO_2_ mobility is higher than the N_2_ mobility in Pebax^®^ membranes due to smaller size and higher condensability of CO_2_ molecules, and the CO_2_-philic ether group in Pebax^®^ polymer chains [14]. The CO_2_ permeance increased with the addition of UiO-66-NH_2_ nanoparticles. The N_2_ permeance increased slightly when the UiO-66-NH_2_ content increased from 0 to 10 wt%. However, when the UiO-66-NH_2_ content was higher than 10 wt%, the N_2_ permeance started to increase significantly, resulting in the decrease in CO_2_/N_2_ ideal selectivity. For instance, when 20 and 50 wt% of UiO-66-NH_2_ was incorporated into the Pebax^®^ 2533 matrix, the CO_2_ permeance increased 9% while the N_2_ permeance increased 25%. Consequently, the CO_2_/N_2_ ideal selectivity was less than that of pure Pebax^®^ 2533 membranes. This can be explained by the severe agglomeration of UiO-66-NH_2_ when large amounts of UiO-66-NH_2_ particles were incorporated into polymeric matrix. Consequently, the non-selective interface defects were formed, resulting in the deterioration of gas separation of Pebax^®^ 2533-UiO-66-NH_2_/PP thin film mixed matrix hollow fiber membranes. The agglomeration of nanoparticles in the mixed matrix membranes have been documented in the literature [14,41,43]. Shen et al. [14] prepared Pebax^®^ 1657 based mixed matrix membranes containing UiO-66 and UiO-66-NH_2_ nanoparticles for CO_2_ separation. It was found that CO_2_/N_2_ selectivity started to decrease due to the filler agglomeration when the UiO-66 and UiO-66-NH_2_ loading is higher than 7.5 and 10 wt%, respectively. Jiao et al. [43] synthesized polyethyleneimine (PEI) modified ZIF-8 and incorporated the PEI-ZIF-8 particle into Pebax^®^ 1657 matrix to prepare mixed matrix membranes for CO_2_/N_2_ separation. It was found that the composite membrane with 5 wt% PEI-ZIF-8 shows the best gas separation performance with CO_2_ permeance equal to 13 GPU and CO_2_/N_2_ selectivity equal to 49. The filler agglomeration occurred resulting in rigidified interface.

### 3.4. Comparison of the Pebax^®^-Based Mixed Matrix Membranes Incorporating Various Nanoparticles in CO_2_/N_2_ Gas Separation

The performance of the prepared Pebax^®^ 2533-UiO-66-NH_2_/PP thin film mixed matrix hollow fiber membranes were compared with Pebax^®^-based mixed matrix membranes containing various types of fillers (Table 1). The gas separation performance of Pebax^®^ 2533-UiO-66-NH_2_/PP thin film mixed matrix hollow fiber membrane containing 10 wt% UiO-66-NH_2_ is comparable with previous reported Pebax^®^-based mixed matrix membranes containing various types of fillers in literature [14,33,40,44,45,46,47,48,49,50,51,52]. The prepared membrane shows a high CO_2_/N_2_ ideal selectivity equal to 37 with a CO_2_ permeance 25.81 GPU at feed pressure 2 bar. The addition of UiO-66-NH_2_ enhances the CO_2_/N_2_ separation performance mainly due to the good interfacial compatibility and the CO_2_-philic nature of UiO-66-NH_2_. Sutrisna et al. [33] fabricated UiO-66-NH_2_/Pebax^®^ 1657 based hollow fiber composite membranes with high CO_2_ permeance equal to 338 GPU and high CO_2_/N_2_ selectivity equal to 57. Their work showed better CO_2_/N_2_ separation performance, which can be attributed to the lower Pebax^®^ 1657 coating solution, and the application of poly [1-(trimethylsilyl) prop-1-yne] (PTMSP) as a gutter layer. The lower coating solution concentration could result in smaller selective layer thickness. The smooth PTMSP gutter layer can prevent the intrusion of Pebax^®^ into pores of support layer, resulting in a thin selective layer. As a result, the prepared UiO-66-NH_2_/Pebax^®^ 1657 based hollow fiber composite membranes showed very high gas separation performance. As it is shown in Table 1, the thin film mixed matrix membranes possess comparable CO_2_/N_2_ selectivity but much higher CO_2_ permeance than that of dense flat sheet mixed matrix membranes. Therefore, the gas separation performances of thin film mixed matrix membranes are better than the dense flat sheet membranes.

## 4. Conclusions

Pebax^®^ 2533-UiO-66-NH_2_/PP mixed matrix thin film hollow fiber membranes were successfully fabricated by using dip coating method. The pre-treatment of PP hollow fibers by dip-coating with 3 wt% Pebax^®^ 2533 solution could smoothen the outer surface of hollow fiber supports, which facilitated the formation of defect-free selective layer. The incorporation of UiO-66-NH_2_ nanoparticles into the Pebax^®^ 2533 coating solution affected the morphology, surface chemistry, and gas separation performance of Pebax^®^ 2533-UiO-66-NH_2_/PP mixed matrix thin film hollow fiber membranes confirmed by SEM analysis, FTIR analysis, and gas permeance measurements, respectively. The aggregation of UiO-66-NH_2_ nanoparticles was observed at higher amounts of UiO-66-NH_2_ nanoparticles in the Pebax^®^ 2533 matrix. The filler aggregation should be tackled by post-synthetic modification of UiO-66-NH_2_ nanoparticles. The CO_2_ permeance increased with the increase of the loading amount of UiO-66 nanoparticles, while the CO_2_/N_2_ ideal gas selectively firstly increased then decreased due to the aggregation of UiO-66 nanoparticles. The Pebax^®^ 2533-UiO-66-NH_2_/PP mixed matrix thin film hollow fiber membranes containing 10 wt% UiO-66 nanoparticles exhibited the best gas separation performance with CO_2_ permeance of 26 GPU and CO_2_/N_2_ selectivity of 37.

## Figures and Tables

**Table 1:** The comparison of gas separation performances of Pebax^®^-based mixed matrix membranes with different fillers.

Support Polymer	Hybrid Coating Material	Filler Content (wt%)	Configuration	Feed Gas	CO_2_ (GPU)	N_2_ (GPU)	CO_2_/N_2_ Selectivity	Ref.
PVDF/PTMSP	UiO-66-NH_2_/Pebax^®^ 1657	50	Hollow fiber	Pure gas	338	5.93	57	[33]
PSF	Fe(DA)/Pebax^®^ 1657	3	Hollow fiber	Pure gas	90.00	1.61	56	[44]
PVDF	ZIF-8/Pebax^®^ 1657	30	Hollow fiber	Pure gas	350.00	10.94	32	[40]
PVDF	GO/Pebax^®^ 1657	0.1	Hollow fiber	Pure gas	415.00	9.65	43	[46]
PSF	GO/Pebax^®^ 1657	0.4	Hollow fiber	Pure gas	28.08	0.66	43	[47]
PAN	ZIF-7/Pebax^®^ 1657	34	Flat sheet	Pure gas	39.00	0.37	105	[45]
PVDF	UiO-66-NH_2_/Pebax^®^ 1657	20	Flat sheet	Pure gas	125 Barrer	-	25	[14]
-	ZIF-8/Pebax^®^ 1657	20	Dense flat sheet	Pure gas	2.80	0.07	41	[48]
-	NH_2_-MIL-53/Pebax^®^ 1657	10	Dense flat sheet	Pure gas	1.60	0.03	55	[49]
-	NaY/Pebax^®^ 1657	10	Dense flat sheet	Pure gas	3.60	0.10	35	[50]
-	ZIF-7/Pebax^®^ 2533	14	Dense flat sheet	Pure gas	198 Barrer	8.74 Barrer	22.6	[19]
-	ZIF-7-NH_2_/Pebax^®^ 2533	14	Dense flat sheet	Pure gas	206 Barrer	7.53 Barrer	27.3	[19]
-	ZIF-8@GO/Pebax^®^ 2533	6	Dense flat sheet	Pure gas	249 Barrer	5.23 Barrer	47.6	[51]
-	Zn/Ni-ZIF-8/Pebax^®^ 2533	10	Dense flat sheet	Pure gas	321 Barrer	7.5 Barrer	42.8	[52]
PP	UiO-66-NH_2_/Pebax^®^ 2533	10	Hollow fiber	Pure gas	25.81	0.69	37	This work

PVDF—polyvinylidene difluoride, PTMSP—poly [1-(trimethylsilyl) prop-1-yne], PSF—polysulfone, PAN—polyacrylonitrile, PP—polypropylene.

## Data Availability

The data presented in this study are available on request from the corresponding author.

## Supplementary Materials

The following are available online at https://www.mdpi.com/article/10.3390/ma14123366/s1, Figure S1: Hollow fiber module for testing gas permeance of hollow fiber membranes (This module is designed by the Membranes and Membrane Techniques Research Group in Nicolaus Copernicus University in Toruń. All the components of this module including housing part, end caps, and ports are purchased from Swagelok), Figure S2: The Zr element mapping and element analysis of Pebax® 2533-UiO-66-NH_2_/PP thin film mixed matrix hollow fiber membranes by EDX. (a) 15 wt% UiO-66-NH_2_, (b) 20 wt% UiO-66-NH_2_, (c) 50 wt% UiO-66-NH_2_, Table S1: The gas permeance and ideal selectivity of thin film hollow fiber membranes fabricated from single concentration of coating solution, Table S2: The thickness of the Pebax/UiO-66-NH2 hybrid layer measured from the top part and bottom part of the prepared mixed matrix thin film hollow fiber membrane. The bottom part is close to the coating solution while the top part is close to the metal holder during the dip-coating process.
