# Supplementary material for: Thin Film Mixed Matrix Hollow Fiber Membrane Fabricated by Incorporation of Amine Functionalized Metal-Organic Framework for CO2/N2 Separation
Source: Materials (Basel). 2021 Jun 17;14(12):3366. doi: 10.3390/ma14123366 (PMC8233894; doi:10.3390/ma14123366)
Supplement: Supplementary file 1 [file materials-14-03366-s001.zip › materials-1244190-supplementary.pdf]

materials

Supplementary Material

Thin Film Mixed Matrix Hollow Fiber Membrane Fabricated by Incorporation of Amine Functionalized Metal-Organic Framework for CO_2/N_2 Separation

Guoqiang Li [1], Wojciech Kujawski [1,2,*], Katarzyna Knozowska [1] and Joanna Kujawa [1]

[1] Department of Physical Chemistry and Physical Chemistry of Polymers, Faculty of Chemistry, Nicolaus Copernicus University in Toruń, 7 Gagarina Street, 87-100 Toruń, Poland
[2] Moscow Engineering Physics Institute, National Research Nuclear University MEPhI, 31 Kashira Hwy, 115409 Moscow, Russia
* Correspondence: kujawski@chem.umk.pl; Tel.: +48-56-6114517

Table S1. The gas permeance and ideal selectivity of thin film hollow fiber membranes fabricated from single concentration of coating solution.

Sample	CO_2 Permeance (GPU)	N_2 Permeance (GPU)	CO_2/N_2 Selectivity
Pebax®-6-1-PP*	62.80 ± 0.59	25.92 ± 0.40	2.4
Pebax®-3-1-PP*	376.46 ± 14.02	234.82 ± 9.23	1.6
Pebax®-3-4-PP*	51.85 ± 1.68	28.16 ± 0.55	1.84

* Pebax®-x-y-PP indicates the selective thin layer was formed from x wt% Pebax® 2533 solution with y layers.

Table S2. The thickness of the Pebax/UiO-66-NH_2 hybrid layer measured from the top part and bottom part of the prepared mixed matrix thin film hollow fiber membrane. The bottom part is close to the coating solution while the top part is close to the metal holder during the dip-coating process.

Membrane	Thickness of Selective Layer (μm)		
	Upper Part	Bottom Part	Average
Pristine Pebax	6.06 ± 0.49	7.61 ± 1.17	6.97 ± 1.19
5 wt% UiO-66-NH_2	3.58 ± 0.66	7.49 ± 1.01	5.36 ± 0.97
10 wt% UiO-66-NH_2	5.58 ± 0.83	8.15 ± 0.86	7.36 ± 1.46
15 wt% UiO-66-NH_2	5.06 ± 0.68	6.35 ± 0.70	5.40 ± 0.88
20 wt% UiO-66-NH_2	6.70 ± 0.33	8.26 ± 1.07	6.82 ± 1.16
50 wt% UiO-66-NH_2	4.76 ± 0.76	8.89 ± 1.83	6.83 ± 2.51

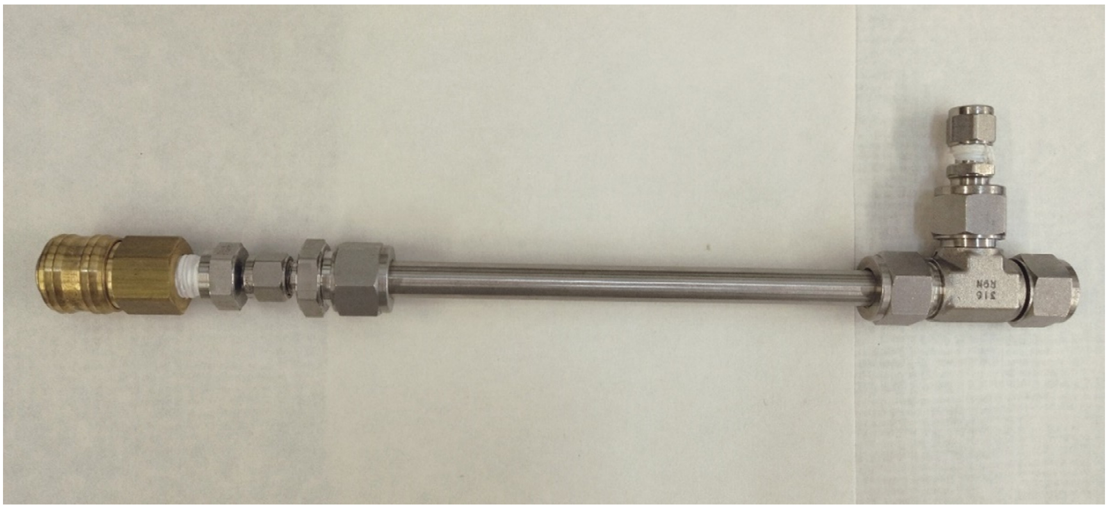

Figure S1. Hollow fiber module for testing gas permeance of hollow fiber membranes (This module is designed by the Membranes and Membrane Techniques Research Group in Nicolaus Copernicus University in Toruń. All the components of this module including housing part, end caps, and ports were purchased from Swagelok, Solon, OH, SUA).

Zr element mapping

Element analysis of UiO-66-NH_2 Pebax® 2533-UiO-66-NH_2/PP membranes

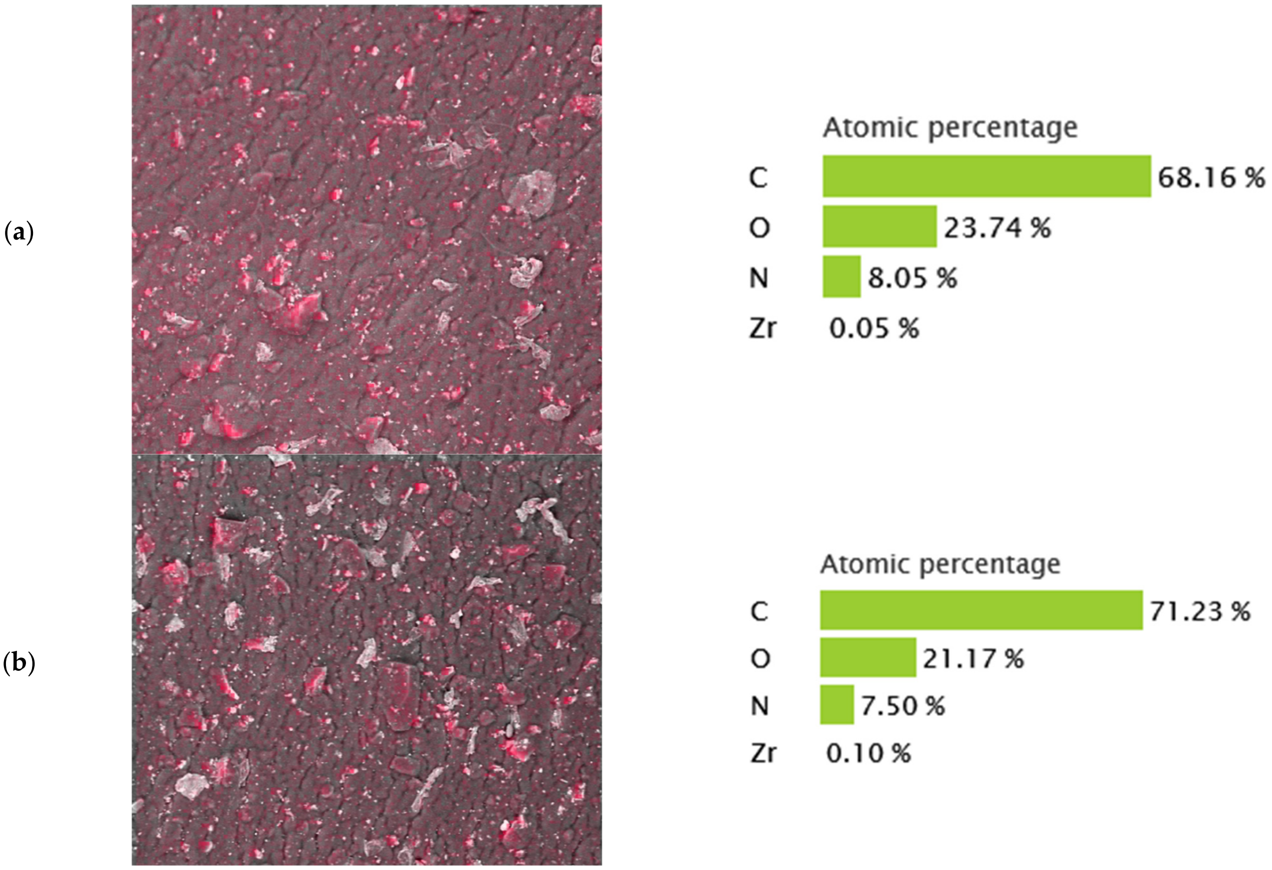

(**c**)

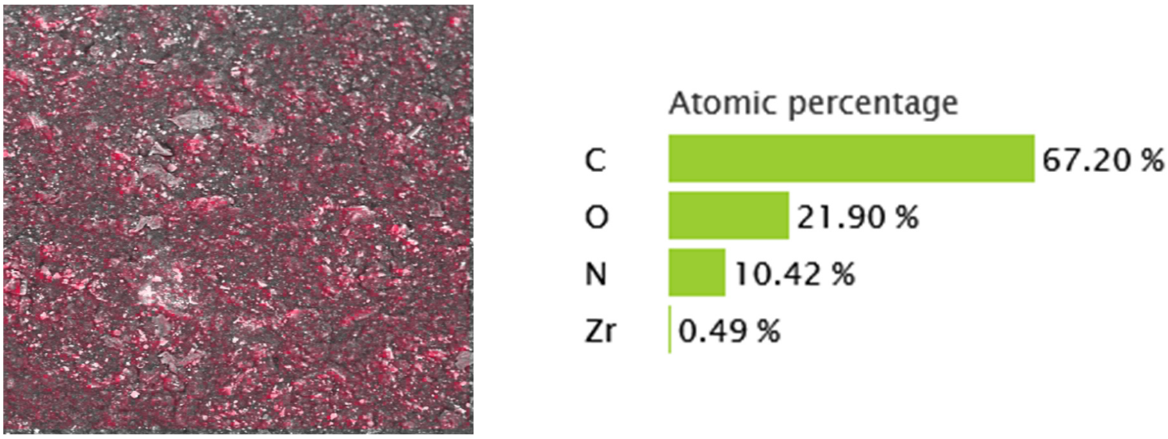

Figure S2. The Zr element mapping and element analysis of Pebax® 2533-UiO-66-NH_2/PP thin film mixed matrix hollow fiber membranes by EDX. (**a**) 15 wt% UiO-66-NH_2, (**b**) 20 wt% UiO-66-NH_2, (**c**) 50 wt% UiO-66-NH_2.
